# Supplementary material for: The Small RNA Universe of Capitella teleta
Source: Front Mol Biosci. 2022 Feb 25;9:802814. doi: 10.3389/fmolb.2022.802814 (PMC8915122; doi:10.3389/fmolb.2022.802814)
Supplement: Supplementary file 1 [file DataSheet1.ZIP › Supplement/known/CAPTEscaffold_135_11090.pdf]

Provisional ID	:	CAPTEscaffold_135_11090
Score total	:	163247.3
Score for star read(s)	:	3.9
Score for read counts	:	163237
Score for mfe	:	1.8
Score for randfold	:	1.6
Score for cons. seed	:	3
Total read count	:	320194
Mature read count	:	317428
Loop read count	:	0
Star read count	:	2766

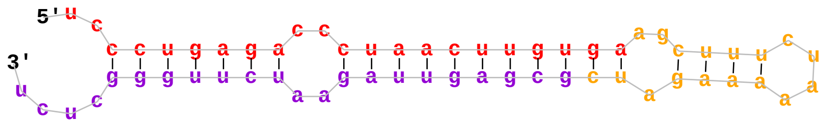

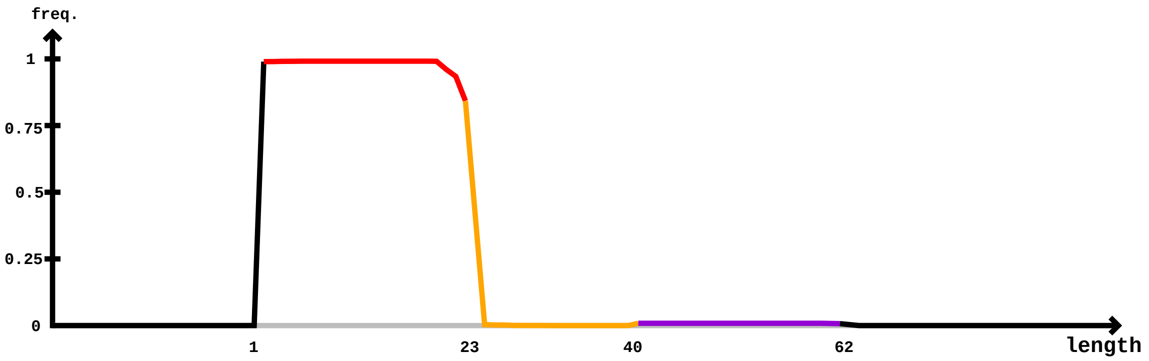

Mature Star

```
5'- aaccacucggcaucucgcacucccugagacccuaacuugugaagcuuucuaaaaagaucgcgaguuagaaucuugggcucugcgaggagcucgauugccgucgaaugaa  -3'  obs
    aaccacucggcaucucgcacucccugagacccuaacuugugaagcuuucuaaaaagaucgcgaguuagaaucuugggcucugcgaggagcucgauugccgucgaaugaa        exp
    aaccacucggcaucucgcacucccugagacccuaacuugugaagcuuucuaaaaagaucgcgaguuagaaucuugggcucugcgaggagcucgauugccgucgaaugaa        known
    ...((.(((((.(((((((...(((((((..((((((((((..((((....)))).))))))))))..)))))))...))))))).)).))).))..............  reads  mm  sample
    ..................Ucucccugagacccuaacuuguga...................................................................  2  1  seq
    ...................cucccugagacccuaacuugu.....................................................................  6  0  seq
    ...................cucccugagacccuaacuugug....................................................................  10  0  seq
    ...................cucccugagacccuaacuugugG...................................................................  2  1  seq
    ...................cucccugagacccuaacuuguga...................................................................  4  0  seq
    ....................ucccugagacccuaacuu.......................................................................  161  0  seq
    ....................Gcccugagacccuaacuu.......................................................................  1  1  seq
    ....................ucccugagacccuaacuA.......................................................................  1  1  seq
    ....................Acccugagacccuaacuu.......................................................................  1  1  seq
    ....................ucccugagaAccuaacuug......................................................................  5  1  seq
    ....................ucccugagaccUuaacuug......................................................................  4  1  seq
    ....................ucccCgagacccuaacuug......................................................................  3  1  seq
    ....................ucccugGgacccuaacuug......................................................................  3  1  seq
    ....................ucccugagacUcuaacuug......................................................................  7  1  seq
    ....................ucccugagacccuaacAug......................................................................  3  1  seq
    ....................ucccugaAacccuaacuug......................................................................  2  1  seq
    ....................ucccugagacccuaacuuA......................................................................  6  1  seq
    ....................ucccugagacccuGacuug......................................................................  18  1  seq
    ....................ucccuAagacccuaacuug......................................................................  12  1  seq
    ....................Gcccugagacccuaacuug......................................................................  11  1  seq
    ....................uUccugagacccuaacuug......................................................................  1  1  seq
    ....................ucccugagCcccuaacuug......................................................................  6  1  seq
    ....................uAccugagacccuaacuug......................................................................  3  1  seq
    ....................uccAugagacccuaacuug......................................................................  1  1  seq
    ....................ucGcugagacccuaacuug......................................................................  1  1  seq
    ....................ucccugagaccAuaacuug......................................................................  3  1  seq
    ....................ucccAgagacccuaacuug......................................................................  10  1  seq
    ....................ucccugagacccuaacuGg......................................................................  4  1  seq
    ....................ucccugagacccuaacuCg......................................................................  1  1  seq
    ....................ucAcugagacccuaacuug......................................................................  2  1  seq
    ....................ucccugagacccuaacuug......................................................................  9519  0  seq
    ....................ucccugagacccuaGcuug......................................................................  1  1  seq
```

```
                    Mature                                  Star
aaccacucggcaucucgcacucccugagacccuaacuugugaagcuuucuaaaaagaucgcgaguuagaaucuugggcucugcgaggagcucgauugccgucgaaugaa
....................ucccugagacccCaacuug......................................................................	2	1	seq
....................ucccuUagacccuaacuug......................................................................	2	1	seq
....................ucccugagGcccuaacuug......................................................................	1	1	seq
....................ucccuCagacccuaacuug......................................................................	2	1	seq
....................ucUcugagacccuaacuug......................................................................	11	1	seq
....................uccUugagacccuaacuug......................................................................	6	1	seq
....................ucccugagacccuaacCug......................................................................	2	1	seq
....................ucccugagaccGuaacuug......................................................................	1	1	seq
....................uGccugagacccuaacuug......................................................................	1	1	seq
....................ucccugagacccuaacuAg......................................................................	8	1	seq
....................Ccccugagacccuaacuug......................................................................	2	1	seq
....................ucccugCgacccuaacuug......................................................................	1	1	seq
....................ucccugagacccuaacuuU......................................................................	5	1	seq
....................ucccugagaUccuaacuug......................................................................	5	1	seq
....................ucccugagacccuaacGug......................................................................	1	1	seq
....................uccGugagacccuaacuug......................................................................	1	1	seq
....................Acccugagacccuaacuug......................................................................	38	1	seq
....................ucccugagacAcuaacuug......................................................................	4	1	seq
....................ucccugaCacccuaacuug......................................................................	1	1	seq
....................ucccugagacAcuaacuugu.....................................................................	3	1	seq
....................Acccugagacccuaacuugu.....................................................................	28	1	seq
....................ucccugaAacccuaacuugu.....................................................................	2	1	seq
....................ucccugagaccGuaacuugu.....................................................................	1	1	seq
....................ucccugagacccGaacuugu.....................................................................	1	1	seq
....................ucccugagacccuGacuugu.....................................................................	7	1	seq
....................ucAcugagacccuaacuugu.....................................................................	3	1	seq
....................ucccugagacccuaacuugA.....................................................................	21	1	seq
....................ucccugagacccuaacuugC.....................................................................	1	1	seq
....................ucccAgagacccuaacuugu.....................................................................	8	1	seq
....................Ncccugagacccuaacuugu.....................................................................	4	1	seq
....................ucccugagacccAaacuugu.....................................................................	1	1	seq
....................ucccugUgacccuaacuugu.....................................................................	1	1	seq
....................Ccccugagacccuaacuugu.....................................................................	1	1	seq
....................ucccugagacccuaacuAgu.....................................................................	2	1	seq
....................ucccugagaAccuaacuugu.....................................................................	3	1	seq
....................ucccugagacccuaacAugu.....................................................................	3	1	seq
....................ucccugagaccUuaacuugu.....................................................................	7	1	seq
....................ucGcugagacccuaacuugu.....................................................................	1	1	seq
....................ucccugagUcccuaacuugu.....................................................................	2	1	seq
....................ucccugagacccuaGcuugu.....................................................................	5	1	seq
....................Gcccugagacccuaacuugu.....................................................................	9	1	seq
....................ucccugagacccuaaAuugu.....................................................................	1	1	seq
....................ucccugagacccuaacuuAu.....................................................................	2	1	seq
....................ucccugagGcccuaacuugu.....................................................................	2	1	seq
....................ucccugagacccuaaUuugu.....................................................................	1	1	seq
....................ucccugagacccuaacuugG.....................................................................	16	1	seq
....................ucccGgagacccuaacuugu.....................................................................	1	1	seq
....................ucccugagacccuaacuugu.....................................................................	8176	0	seq
....................ucccugagCcccuaacuugu.....................................................................	4	1	seq
....................ucccugagaUccuaacuugu.....................................................................	2	1	seq
....................ucccugagacUcuaacuugu.....................................................................	37	1	seq
....................ucccCgagacccuaacuugu.....................................................................	3	1	seq
....................ucccuAagacccuaacuugu.....................................................................	9	1	seq
....................ucUcugagacccuaacuugu.....................................................................	4	1	seq
....................uccGugagacccuaacuugu.....................................................................	1	1	seq
....................ucccugagaccAuaacuugu.....................................................................	3	1	seq
....................uccUugagacccuaacuugu.....................................................................	6	1	seq
....................ucccugaCacccuaacuugu.....................................................................	1	1	seq
....................ucccugagacccCaacuugu.....................................................................	1	1	seq
....................ucccuCagacccuaacuugu.....................................................................	3	1	seq
....................ucccugagacGcuaacuugu.....................................................................	1	1	seq
....................uAccugagacccuaacuugu.....................................................................	5	1	seq
....................uNccugagacccuaacuugu.....................................................................	2	1	seq
....................ucccugagacccuaacAugug....................................................................	16	1	seq
....................ucccugagacccuaGcuugug....................................................................	4	1	seq
....................uAccugagacccuaacuugug....................................................................	8	1	seq
....................ucccugagacccuaacGugug....................................................................	2	1	seq
....................Ncccugagacccuaacuugug....................................................................	10	1	seq
....................Acccugagacccuaacuugug....................................................................	108	1	seq
....................ucccugagCcccuaacuugug....................................................................	11	1	seq
```

```
                    Mature                Star
aaccacucggcaucucgcacucccugagacccuaacuugugaagcuuucuaaaaagaucgcgaguuagaaucuugggcucugcgaggagcucgauugccgucgaaugaa
....................ucccugagacccGaacuugug....................................................................	1	1	seq
....................ucccugagacccuaUcuugug....................................................................	3	1	seq
....................ucccugagacccuaaGuugug....................................................................	3	1	seq
....................ucccAgagacccuaacuugug....................................................................	32	1	seq
....................ucccugagacccuaacuugGg....................................................................	7	1	seq
....................ucccugagacccuaaAuugug....................................................................	2	1	seq
....................ucccugagacccuaacuuAug....................................................................	6	1	seq
....................ucccuUagacccuaacuugug....................................................................	2	1	seq
....................ucccugagacccuaacuuguA....................................................................	32	1	seq
....................uccUugagacccuaacuugug....................................................................	13	1	seq
....................ucccugagUcccuaacuugug....................................................................	3	1	seq
....................ucccugagacccuaacuugAg....................................................................	9	1	seq
....................ucccugaUacccuaacuugug....................................................................	1	1	seq
....................uUccugagacccuaacuugug....................................................................	3	1	seq
....................ucccugagGcccuaacuugug....................................................................	2	1	seq
....................ucccugagacccAaacuugug....................................................................	6	1	seq
....................ucccugagaUccuaacuugug....................................................................	1	1	seq
....................ucccugagacccuaacuugCg....................................................................	2	1	seq
....................ucccugagacccuGacuugug....................................................................	9	1	seq
....................ucccuAagacccuaacuugug....................................................................	33	1	seq
....................uccGugagacccuaacuugug....................................................................	1	1	seq
....................Gcccugagacccuaacuugug....................................................................	12	1	seq
....................Ccccugagacccuaacuugug....................................................................	2	1	seq
....................ucccugagacAcuaacuugug....................................................................	19	1	seq
....................uccAugagacccuaacuugug....................................................................	7	1	seq
....................ucccugCgacccuaacuugug....................................................................	1	1	seq
....................ucccGgagacccuaacuugug....................................................................	3	1	seq
....................ucccugagacccuaacuuCug....................................................................	4	1	seq
....................ucccugagacccCaacuugug....................................................................	3	1	seq
....................ucccugagaccAuaacuugug....................................................................	8	1	seq
....................ucccuCagacccuaacuugug....................................................................	3	1	seq
....................ucccugaAacccuaacuugug....................................................................	4	1	seq
....................ucccugaCacccuaacuugug....................................................................	2	1	seq
....................ucccugagacccuaacuuguU....................................................................	9	1	seq
....................ucccugagaccUuaacuugug....................................................................	13	1	seq
....................ucccugagacccuaacuAgug....................................................................	9	1	seq
....................ucccugagacccuaacCugug....................................................................	2	1	seq
....................ucccugagacccuUacuugug....................................................................	1	1	seq
....................ucccugagacccuaacuugug....................................................................	28833	0	seq
....................ucUcugagacccuaacuugug....................................................................	29	1	seq
....................ucccugagacccuaacuuguC....................................................................	3	1	seq
....................ucccugagacccuaacuGgug....................................................................	2	1	seq
....................ucccugUgacccuaacuugug....................................................................	1	1	seq
....................ucccugagaAccuaacuugug....................................................................	12	1	seq
....................ucccugGgacccuaacuugug....................................................................	4	1	seq
....................ucAcugagacccuaacuugug....................................................................	3	1	seq
....................ucccugagacccuaaUuugug....................................................................	4	1	seq
....................ucccugagaGccuaacuugug....................................................................	1	1	seq
....................ucccCgagacccuaacuugug....................................................................	7	1	seq
....................ucccugagacccuaacuCgug....................................................................	3	1	seq
....................ucccugagacUcuaacuugug....................................................................	42	1	seq
....................ucccugagacccuaacuugAga...................................................................	93	1	seq
....................uccAugagacccuaacuuguga...................................................................	35	1	seq
....................ucUcugagacccuaacuuguga...................................................................	174	1	seq
....................ucccugagacccuaacuuguUa...................................................................	14	1	seq
....................ucccugagacccuGacuuguga...................................................................	95	1	seq
....................ucccuCagacccuaacuuguga...................................................................	15	1	seq
....................ucccugagacccuaacuugCga...................................................................	24	1	seq
....................ucccuAagacccuaacuuguga...................................................................	216	1	seq
....................ucccugagaNccuaacuuguga...................................................................	2	1	seq
....................ucccugUgacccuaacuuguga...................................................................	12	1	seq
....................ucGcugagacccuaacuuguga...................................................................	6	1	seq
....................ucccGgagacccuaacuuguga...................................................................	18	1	seq
....................ucccugagacccuaGcuuguga...................................................................	20	1	seq
....................ucccugagacccuaacuugugG...................................................................	30634	1	seq
....................ucccugagacccuaacGuguga...................................................................	8	1	seq
....................ucccugagaccGuaacuuguga...................................................................	12	1	seq
....................ucccugagacccuaCcuuguga...................................................................	2	1	seq
....................ucccAgagacccuaacuuguga...................................................................	258	1	seq
....................ucccugagCcccuaacuuguga...................................................................	101	1	seq
```

```
                    Mature                                 Star
aaccacucggcaucucgcacucccugagacccuaacuugugaagcuuucuaaaaagaucgcgaguuagaaucuugggcucugcgaggagcucgauugccgucgaaugaa
....................ucccugagacccuUacuuguga...................................................................  3       1  seq
....................uUccugagacccuaacuuguga...................................................................  43      1  seq
....................ucccugagacNcuaacuuguga...................................................................  1       1  seq
....................ucccugGgacccuaacuuguga...................................................................  61      1  seq
....................uAccugagacccuaacuuguga...................................................................  39      1  seq
....................ucccugagacccuaacuuUuga...................................................................  8       1  seq
....................ucccugagacccuaacCuguga...................................................................  11      1  seq
....................ucccugaUacccuaacuuguga...................................................................  8       1  seq
....................ucccugaAacccuaacuuguga...................................................................  36      1  seq
....................ucccugagacccuaacuAguga...................................................................  94      1  seq
....................ucccuUagacccuaacuuguga...................................................................  16      1  seq
....................ucccugagacccuaacuugugC...................................................................  14      1  seq
....................ucccugagacGcuaacuuguga...................................................................  9       1  seq
....................ucccugagacccuaUcuuguga...................................................................  18      1  seq
....................ucccugagacccuaacuuguCa...................................................................  13      1  seq
....................ucccugagacUcuaacuuguga...................................................................  3288    1  seq
....................ucccugagacccuaacuugugU...................................................................  614     1  seq
....................Ncccugagacccuaacuuguga...................................................................  67      1  seq
....................ucccugCgacccuaacuuguga...................................................................  4       1  seq
....................ucccugagGcccuaacuuguga...................................................................  32      1  seq
....................ucccugagacccAaacuuguga...................................................................  45      1  seq
....................uccUugagacccuaacuuguga...................................................................  90      1  seq
....................ucccugagacccuaacuuAuga...................................................................  30      1  seq
....................ucccugagacccuaacuuguga...................................................................  215649  0  seq
....................ucccugagacccuaaAuuguga...................................................................  13      1  seq
....................ucccugagacccuaaUuuguga...................................................................  24      1  seq
....................ucccugagaAccuaacuuguga...................................................................  87      1  seq
....................ucccugagacccuaacAuguga...................................................................  78      1  seq
....................ucccugagacccuaacuuCuga...................................................................  11      1  seq
....................Ccccugagacccuaacuuguga...................................................................  85      1  seq
....................ucccugagacccuaacuuguAa...................................................................  86      1  seq
....................ucccugagacccuaacuGguga...................................................................  10      1  seq
....................ucccugagaGccuaacuuguga...................................................................  12      1  seq
....................ucccugagaUccuaacuuguga...................................................................  27      1  seq
....................uccGugagacccuaacuuguga...................................................................  7       1  seq
....................ucccugaCacccuaacuuguga...................................................................  13      1  seq
....................ucccugagaccUuaacuuguga...................................................................  85      1  seq
....................ucccugagacccCaacuuguga...................................................................  19      1  seq
....................ucccugagacccGaacuuguga...................................................................  16      1  seq
....................ucccugagacccuaacuuguNa...................................................................  2       1  seq
....................uNccugagacccuaacuuguga...................................................................  5       1  seq
....................uGccugagacccuaacuuguga...................................................................  3       1  seq
....................ucccugagacccuaaGuuguga...................................................................  9       1  seq
....................Gcccugagacccuaacuuguga...................................................................  165     1  seq
....................ucccugagaccAuaacuuguga...................................................................  78      1  seq
....................ucccugagacccuaacuugGga...................................................................  18      1  seq
....................ucccugagUcccuaacuuguga...................................................................  21      1  seq
....................ucccugagacAcuaacuuguga...................................................................  159     1  seq
....................ucAcugagacccuaacuuguga...................................................................  48      1  seq
....................ucccugagacccuaacuCguga...................................................................  33      1  seq
....................Acccugagacccuaacuuguga...................................................................  831     1  seq
....................ucccCgagacccuaacuuguga...................................................................  51      1  seq
....................ucccugUgacccuaacuugugaa..................................................................  2       1  seq
....................ucccugGgacccuaacuugugaa..................................................................  1       1  seq
....................uAccugagacccuaacuugugaa..................................................................  5       1  seq
....................ucccugagaUccuaacuugugaa..................................................................  2       1  seq
....................ucUcugagacccuaacuugugaa..................................................................  11      1  seq
....................ucccugaAacccuaacuugugaa..................................................................  1       1  seq
....................ucccugagacccuaacuugCgaa..................................................................  2       1  seq
....................ucccugagaccUuaacuugugaa..................................................................  6       1  seq
....................ucccugaUacccuaacuugugaa..................................................................  1       1  seq
....................ucccugagacccuaacCugugaa..................................................................  1       1  seq
....................Ccccugagacccuaacuugugaa..................................................................  1       1  seq
....................ucccugagacccuaacAugugaa..................................................................  4       1  seq
....................ucccugagacUcuaacuugugaa..................................................................  8       1  seq
....................ucccugagacccuaGcuugugaa..................................................................  1       1  seq
....................ucccAgagacccuaacuugugaa..................................................................  11      1  seq
....................ucccugagacccAaacuugugaa..................................................................  4       1  seq
....................ucccugaCacccuaacuugugaa..................................................................  2       1  seq
....................ucccugagacccuaacuugugUa..................................................................  3       1  seq
```

```
                    Mature                                 Star
aaccacucggcaucucgcacucccugagacccuaacuugugaagcuuucuaaaaagaucgcgaguuagaaucuugggcucugcgaggagcucgauugccgucgaaugaa
....................ucccugagacccuaUcuugugaa..................................................................  1      1  seq
....................ucccuAagacccuaacuugugaa..................................................................  10     1  seq
....................ucccugagacccuaacuuAugaa..................................................................  1      1  seq
....................ucccugagacccuGacuugugaa..................................................................  11     1  seq
....................Gcccugagacccuaacuugugaa..................................................................  7      1  seq
....................ucccugagGcccuaacuugugaa..................................................................  2      1  seq
....................uNccugagacccuaacuugugaa..................................................................  2      1  seq
....................Ncccugagacccuaacuugugaa..................................................................  4      1  seq
....................ucccugagacccuaacuuguAaa..................................................................  11     1  seq
....................ucccugagaAccuaacuugugaa..................................................................  5      1  seq
....................uUccugagacccuaacuugugaa..................................................................  2      1  seq
....................ucccugagacccuaacuCgugaa..................................................................  1      1  seq
....................ucccugagacccuaacuugugaG..................................................................  164    1  seq
....................uccAugagacccuaacuugugaa..................................................................  1      1  seq
....................ucccugagacccuaaUuugugaa..................................................................  2      1  seq
....................ucccCgagacccuaacuugugaa..................................................................  4      1  seq
....................ucAcugagacccuaacuugugaa..................................................................  1      1  seq
....................ucccugagacccuaacuGgugaa..................................................................  1      1  seq
....................ucccugagaccAuaacuugugaa..................................................................  4      1  seq
....................ucccuCagacccuaacuugugaa..................................................................  2      1  seq
....................Acccugagacccuaacuugugaa..................................................................  45     1  seq
....................ucccugagacccCaacuugugaa..................................................................  4      1  seq
....................ucccugagCcccuaacuugugaa..................................................................  3      1  seq
....................uccUugagacccuaacuugugaa..................................................................  3      1  seq
....................ucccugagacccGaacuugugaa..................................................................  1      1  seq
....................ucccugagacccuaacuugugaC..................................................................  94     1  seq
....................ucccugagacAcuaacuugugaa..................................................................  10     1  seq
....................ucccugagacccuaacuugugaa..................................................................  10502  0  seq
....................ucccugagacccuaacuugugaU..................................................................  3315   1  seq
....................ucccugagacccuaacuugAgaa..................................................................  4      1  seq
....................ucccugagacccuaacuugugGa..................................................................  2      1  seq
....................ucccugagacccuaacuAgugaa..................................................................  5      1  seq
....................ucccugagacccuaacuugugaaA.................................................................  241    1  seq
....................ucccugagacccuaacuugugaaU.................................................................  164    1  seq
....................ucccugagacccuaacuugugaUg.................................................................  4      1  seq
....................ucccugagacccuaacuugugaaC.................................................................  14     1  seq
....................ucccugagacccuaacuugugaagc................................................................  3      0  seq
....................ucccugagacccuaacuAgugaagcu...............................................................  1      1  seq
....................ucccAgagacccuaacuugugaagcu...............................................................  1      1  seq
....................uAccugagacccuaacuugugaagcu...............................................................  1      1  seq
....................Gcccugagacccuaacuugugaagcu...............................................................  1      1  seq
....................ucccugagacUcuaacuugugaagcu...............................................................  1      1  seq
....................ucccugagacccuaacuugugaagcu...............................................................  493    0  seq
....................ucccugagacccuaacuuUugaagcu...............................................................  1      1  seq
....................ucccugagacccuaacuGgugaagcu...............................................................  1      1  seq
....................ucccugagCcccuaacuugugaagcuu..............................................................  1      1  seq
....................ucccugagacccuaacuugugaagcuA..............................................................  1      1  seq
....................ucccugagacccuaacuugugaagcuu..............................................................  85     0  seq
....................ucccugagacccuaacuugugaagcuuu.............................................................  6      0  seq
....................ucccugagacccuaacuugugaagcuuuU............................................................  1      1  seq
....................ucccugagacccuaacuugugaagcuAucu...........................................................  1      1  seq
....................Acccugagacccuaacuugugaagcuuucu...........................................................  2      1  seq
....................ucccugagacccuaacuugugaagcuuucu...........................................................  86     0  seq
....................ucccugagacccuaacuugugaagcuGucu...........................................................  1      1  seq
.....................Uccugagacccuaacuug......................................................................  2      1  seq
.....................Uccugagacccuaacuugu.....................................................................  1      1  seq
.....................cccugagacccuaacuugug....................................................................  1      0  seq
.....................Accugagacccuaacuuguga...................................................................  2      1  seq
.....................Uccugagacccuaacuuguga...................................................................  16     1  seq
.....................cccugagacccuaacuugugG...................................................................  1      1  seq
.....................cccugagacccuaacuuguga...................................................................  3      0  seq
.....................cccugagacccuaacuugugaa..................................................................  31     0  seq
.....................cccugagacccuaacuugugaC..................................................................  1      1  seq
......................ccugagacccuaacuugu.....................................................................  1      0  seq
......................ccugagacccuaacuugug....................................................................  12     0  seq
......................ccugagacccAaacuugug....................................................................  1      1  seq
......................ccAgagacccuaacuugug....................................................................  3      1  seq
......................ccugagacccAaacuuguga...................................................................  1      1  seq
......................ccugagaccUuaacuuguga...................................................................  1      1  seq
......................ccAgagacccuaacuuguga...................................................................  10     1  seq
```

Mature **Star**

```
aaccacucggcaucucgcacucccugagacccuaacuugugaagcuuucuaaaaagaucgcgaguuagaaucuugggcucugcgaggagcucgauugccgucgaaugaa
......................ccugagacccuaacuuguga...................................................................	221	0	seq
......................ccugagacccuaacuugugG...................................................................	28	1	seq
......................Ucugagacccuaacuuguga...................................................................	3	1	seq
......................ccugagacccuaUcuuguga...................................................................	1	1	seq
......................ccGgagacccuaacuuguga...................................................................	1	1	seq
......................ccugagacccuaacuugugaU..................................................................	3	1	seq
......................ccugagacccuaacuugugaa..................................................................	4	0	seq
......................ccugagacccAaacuugugaagc................................................................	1	1	seq
.......................cugagacccuaacuugugG...................................................................	1	1	seq
.......................cugagacccuaacuuguga...................................................................	13	0	seq
........................ugagacccuaacuuguga...................................................................	128	0	seq
........................ugagaccUuaacuuguga...................................................................	1	1	seq
........................ugagacccuaacuugugG...................................................................	18	1	seq
........................Agagacccuaacuuguga...................................................................	1	1	seq
........................ugagacccuaacuugugaU..................................................................	1	1	seq
........................ugagacccuaacuugugaagc................................................................	3	0	seq
........................ugagacccuaacuugugaagcuu..............................................................	3	0	seq
..........................................................cgcgaguuagaaucuugggcu..............................	12	0	seq
..........................................................cgcgaguuagaaucuugggcuc.............................	7	0	seq
..........................................................cgcgaguuagaaucuugggcucu............................	3	0	seq
...........................................................gcgaguuagaaucuugggc...............................	10	0	seq
...........................................................gcgaguuagaaucuugggcG..............................	2	1	seq
...........................................................gcgaguuagaaucuugggcu..............................	182	0	seq
...........................................................gcgaguuGgaaucuugggcu..............................	40	1	seq
...........................................................gcgaguuagaaucuugggcuc.............................	185	0	seq
...........................................................gcgaguuagaaucuugggcuA.............................	1	1	seq
...........................................................gcgaguuagaaucuuggAcuc.............................	1	1	seq
...........................................................gcgaguuagaGucuugggcuc.............................	14	1	seq
...........................................................gcgaguuagaaucuugggcuU.............................	6	1	seq
...........................................................gcgaguuagaaucCugggcucu............................	1	1	seq
...........................................................gcgaguAagaaucuugggcucu............................	3	1	seq
...........................................................gcgaguuagaGucuugggcucu............................	25	1	seq
...........................................................gcgaguuagaaucuugggcucC............................	1	1	seq
...........................................................gcgaguuagaaucuuAggcucu............................	5	1	seq
...........................................................gcgaguuagaaucuugggGucu............................	1	1	seq
...........................................................Ucgaguuagaaucuugggcucu............................	8	1	seq
...........................................................gcgaguuagaaucuugggcucu............................	2184	0	seq
...........................................................gcgagCuagaaucuugggcucu............................	1	1	seq
...........................................................gcgaguuaAaaucuugggcucu............................	2	1	seq
...........................................................Acgaguuagaaucuugggcucu............................	1	1	seq
...........................................................gcgaguuagaaucuAgggcucu............................	1	1	seq
...........................................................gcAaguuagaaucuugggcucu............................	5	1	seq
...........................................................gcgaguuagaaucuugggcucG............................	3	1	seq
...........................................................gcgaguuagaaucAugggcucu............................	1	1	seq
...........................................................gcgaguuagaaucuugggcucA............................	2	1	seq
...........................................................gcgaguuGgaaucuugggcucu............................	10	1	seq
...........................................................gcCaguuagaaucuugggcucu............................	1	1	seq
...........................................................gcgaguuagaauAuugggcucu............................	1	1	seq
...........................................................Ncgaguuagaaucuugggcucu............................	1	1	seq
...........................................................gcgaguuagaaAcuugggcucu............................	2	1	seq
...........................................................gcgaguuagaaucuugggcucuA...........................	5	1	seq
...........................................................gcgaguuagaaucuugggcucuU...........................	37	1	seq
............................................................cgaguuagaaucuugggcucu............................	1	0	seq
.............................................................gaguuagaaucuugggcucu............................	1	0	seq
```
